# Highly Pathogenic Avian Influenza H5N1, Thailand, 2004

**DOI:** 10.3201/eid1111.050608

**Published:** 2005-11

**Authors:** Thanawat Tiensin, Prasit Chaitaweesub, Thaweesak Songserm, Arunee Chaisingh, Wirongrong Hoonsuwan, Chantanee Buranathai, Tippawon Parakamawongsa, Sith Premashthira, Alongkorn Amonsin, Marius Gilbert, Mirjam Nielen, Arjan Stegeman

**Affiliations:** * Department of Livestock Development, Bangkok, Thailand; † Utrecht University, Utrecht, the Netherlands; ‡ Kasetsart University, Nakhon Pathom, Thailand; § National Institute of Animal Health, Bangkok, Thailand; ¶ Chulalongkorn University, Bangkok, Thailand; # Free University of Brussels, Brussels, Belgium

**Keywords:** Influenza, avian influenza, epidemiology, poultry, control measures, Thailand, synopsis

## Abstract

In January 2004, highly pathogenic avian influenza (HPAI) virus of the H5N1 subtype was first confirmed in poultry and humans in Thailand. Control measures, e.g., culling poultry flocks, restricting poultry movement, and improving hygiene, were implemented. Poultry populations in 1,417 villages in 60 of 76 provinces were affected in 2004. A total of 83% of infected flocks confirmed by laboratories were backyard chickens (56%) or ducks (27%). Outbreaks were concentrated in the Central, the southern part of the Northern, and Eastern Regions of Thailand, which are wetlands, water reservoirs, and dense poultry areas. More than 62 million birds were either killed by HPAI viruses or culled. H5N1 virus from poultry caused 17 human cases and 12 deaths in Thailand; a number of domestic cats, captive tigers, and leopards also died of the H5N1 virus. In 2005, the epidemic is ongoing in Thailand.

## Abstract 2

Early detection and control curtail outbreaks.

Highly pathogenic avian influenza (HPAI) is a devastating disease in poultry; it is associated with a high death rate and disrupts poultry production and trade (*1**,**2*). HPAI viruses may be transmitted from birds to humans (*3**,**4*), and they are a potential source of future human influenza pandemics (*5*). HPAI outbreaks were relatively rare until 1990 but occurred in many countries in the last decade (*1*). In Asia, since the HPAI H5N1 epidemic in Hong Kong in 1997, HPAI viruses have been isolated continuously through routine surveillance in Hong Kong (*6**,**7*), South Korea (*8*), and China (*9**–**11*). In Thailand, no evidence of HPAI infection was recorded before 2004 (*12*). In 2003 and 2004, HPAI H5N1 outbreaks were reported in several Asian countries (South Korea, Vietnam, Japan, Thailand, Cambodia, Hong Kong, Laos, Indonesia, China, and Malaysia) (*1**,**13*), and these outbreaks were not easily halted (*11**,**14*). Furthermore, H5N1 viruses crossed from birds to humans and caused 116 laboratory-confirmed cases in Vietnam, Thailand, Cambodia, and Indonesia with 60 deaths (as of September 29, 2005) (*11**,**15*). We describe epidemiologic features of the HPAI H5N1 epidemic in Thailand in 2004, with focus on introduction of the virus, distribution of disease in Thai poultry, control measures, and consequences.

## Poultry in Thailand and HPAI Virus Introduction

**Figure 1:**
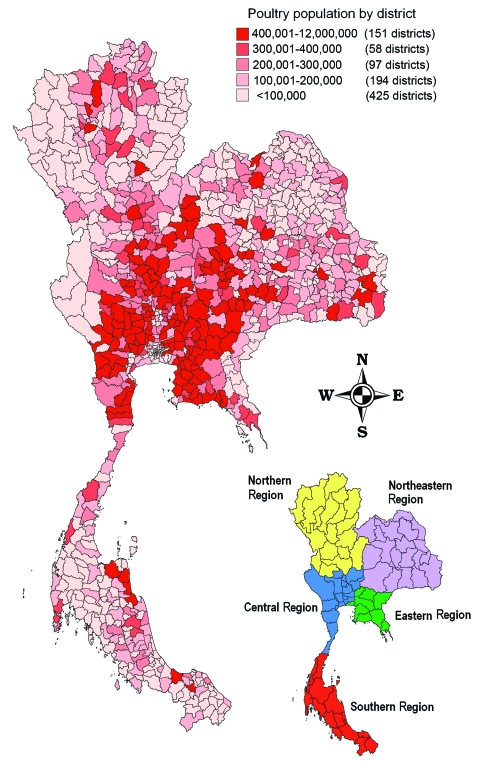
Distribution of poultry population in Thailand in 2003.

Before 2004, Thailand was among the world's major poultry exporters and produced ≈1 billion chickens per year (*16*); >400,000 persons were employed in the poultry industry (*17*). Aside from commercial hybrid broilers and layers, backyard poultry are raised for food in most villages (*18*) (Table 1). The poultry population is concentrated in the Central and Eastern Regions of Thailand (Figure 1). Table 2 categorizes Thai poultry production into 4 sectors on the basis of farm management, biosecurity, and market orientation (*14*).

**Table 1:** Poultry population categorized by geographic region in Thailand in 2003

Poultry population	North	Central	East	South	Northeast	Total	RR (95% CI)*
Backyard chickens							
Birds	18,067,529	9,312,042	3,880,535	6,280,375	25,551,093	63,091,574	
Flocks	543,793	143,829	81,804	241,886	1,125,352	2,136,664	
Infected flocks	491	296	107	31	94	1,019	
Incidence, %	0.0903	0.2058	0.1308	0.0128	0.0084	0.0477	1.0
Layers							
Birds	2,288,485	7,682,667	8,304,081	2,113,035	3,924,255	24,312,523	
Flocks	4,209	6,396	3,941	7,666	14,264	36,476	
Infected flocks	29	42	14	1	6	92	
Incidence, %	0.6890	0.6567	0.3552	0.0130	0.0421	0.2522	5.3 (4.4–6.4)
Broilers							
Birds	12,442,797	70,414,281	53,681,571	6,565,161	22,210,976	165,314,786	
Flocks	4,588	6,242	6,507	6,166	22,274	45,777	
Infected flocks	44	54	8	3	2	111	
Incidence, %	0.9590	0.8651	0.1229	0.0487	0.0090	0.2425	5.1 (4.3–6.1)
Ducks							
Birds	2,567,666	8,026,701	6,110,934	1,777,466	5,317,325	23,800,092	
Flocks	58,606	33,607	17,917	95,216	478,483	683,829	
Infected flocks	85	355	29	9	13	491	
Incidence, %	0.1450	1.0563	0.1619	0.0095	0.0027	0.0718	1.5 (1.3–1.7)
Quails							
Birds	199,357	2,920,216	189,342	302,291	81,597	3,692,803	
Flocks	147	324	114	1,797	211	2,593	
Infected flocks	12	26	–	1	1	40	
Incidence, %	8.1633	8.0247	0	0.0556	0.4739	1.5426	32.4 (26.5–39.5)
Geese							
Birds	8,098	154,723	101,465	9,980	34,401	308,667	
Flocks	1,650	1,870	923	2,596	7,646	14,685	
Infected flocks	4	8	3	–	1	16	
Incidence, %	0.2424	0.4278	0.3250	0	0.0131	0.1090	2.3 (1.4–3.7)
Other							
Infected flocks	10	14	6	–	10	40	
Total							
Birds	35,573,932	98,510,630	72,267,928	17,048,308	57,119,647	280,520,445	
Flocks†	612,993	192,268	111,206	355,327	1,648,230	2,920,024	
Infected flocks‡	665	781	161	45	117	1,769	
Incidence, %	0.1085	0.4062	0.1448	0.0127	0.0071	0.0606	
RR (95% CI)	1.0	3.7 (3.4–4.1)	1.3 (1.1–1.6)	0.1 (0.01–0.15)	0.05 (0.06–0.08)		

*Relationship between cumulative incidence and relative risk (RR) of influenza H5N1 epidemic. Cumulative incidence of infected flocks of backyard chickens and the Central Region were assigned an RR of 1.0. †Included only the number of flocks of backyard chickens, layers, broilers, ducks, quails, and geese. Some flocks are mixed types of poultry on site. ‡Included only the number of infected flocks of backyard chickens, layers, broilers, ducks, quails, and geese.

**Table 2:** Poultry production system in Thailand*

Poultry production	Biosecurity	Market orientation	Example
Sector 1	High	Commercial	Industrial integrated system: all components of the production chain (e.g., hatchery, feedmill, poultry farm, slaughterhouse, processing plant, transportation) owned by company with strictly implemented procedures for biosecurity
Sector 2	Moderate to high	Commercial	Semivertical integrated system (or contract farming system): poultry houses owned by the farmer but chicks, feed, and veterinary service supplied by private company. Birds kept indoors with basic physical barriers and hygiene to prevent contact with other animals
Sector 3	Low	Commercial, local, or live-bird market	Layer farm with caged birds in open sheds or free-roaming birds that spend time outside the shed
Sector 4	None	Local	Village or backyard poultry: birds freely roam the village around people and other animals, including cockfighting

*Source: Food and Agriculture Organization (*14*).

In late 2003, poultry farms in the Central and Northern Regions of Thailand experienced large-scale die-offs (*19**–**22*). Beginning in mid-December 2003, H5N1 outbreaks were reported in South Korea, Vietnam, and Japan. Meanwhile, Vietnam confirmed the first human death from H5N1 (*13*). In December 2003, a nationwide surveillance program was initiated to detect human cases in Thailand (*22*). Subsequently, the surveillance program was strenuously implemented for poultry in mid-January 2004. Cloacal swabs were collected from poultry flocks throughout Thailand, and all samples were tested for avian influenza by virus isolation (*2*) at national and regional laboratories of the Thai Department of Livestock Development (DLD).

On January 23, 2004, the Thai national reference laboratory (National Institute of Animal Health [NIAH]) officially confirmed the presence of an H5 HPAI virus in a layer chicken farm in Suphanburi Province (*13*). The route by which this virus was introduced could not be traced. The virus was characterized as the H5N1 subtype (*13*), a member of the 2000 avian influenza lineage; most of its genetic sequences were closely related to influenza A/Duck/China/E319.2/03 (*23*); it belonged to genotype Z (*11*). That same day, the Thai Ministry of Public Health (MOPH) announced 2 laboratory-confirmed cases of H5N1 virus in children from Suphanburi and Kanchanaburi Provinces; the children eventually died (*24**,**25*).

## Spread of the Epidemic

**Figure 2:**
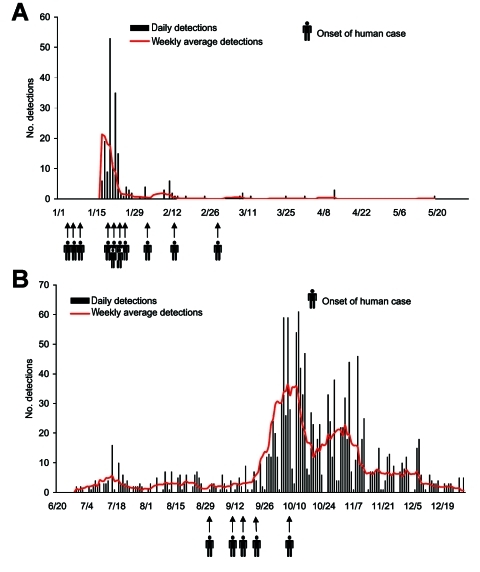
Epidemic curve of the confirmed highly pathogenic avian influenza H5N1 outbreaks in poultry in Thailand by date of notification. A) January–May 2004. B) July–December 2004.

The onset of H5N1 human cases (*22*) showed that the H5N1 virus was already introduced into Thailand by the end of 2003 (Figure 2), before the first identification of the virus. In addition, the 149 reported outbreaks in poultry in 144 villages in 32 of the 76 provinces during the first week of the epidemic indicated that the virus had been widespread throughout the country. The epidemics in Thailand took place in 2 distinct periods, January–May 2004 (termed P1 or the first wave) and July–December 2004 (termed P2 or the second wave) (Figure 2). The epidemic is ongoing in Thailand in 2005, but the current analysis includes only outbreaks from January to December 2004.

From January to May 2004, HPAI infections were detected in 188 villages in 42 of 76 provinces throughout Thailand (Table 3). The outbreaks occurred in all parts of Thailand but particularly in the Central, the southern part of the Northern, and the Eastern Regions. The last outbreak of the first wave was reported on May 24, 2004, from a layer farm in Chiangmai Province (*13*).

**Table 3:** Number of detections of highly pathogenic avian influenza H5N1 outbreaks in each administrative division during epidemic in Thailand, 2004 (n = 1,685 flocks, record with missing data excluded)

Administrative division	No. detections (Jan–May)	No. detections (Jul–Dec)	Total (Jan–Dec)*
Province (N = 76)	42	51	60
District (N = 926)	89	264	305
Subdistrict (N = 7,409)	146	781	903
Village (N = 71,864)	188	1,243	1,417

*Some HPAI outbreaks during P2 (Jul–Dec) occurred repeatedly in the same administrative division as during P1 (Jan–May).

**Figure 3:**
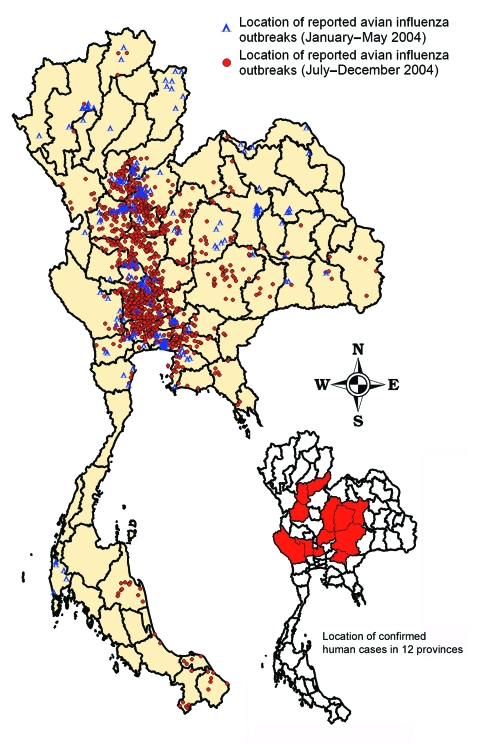
Distribution of reported highly pathogenic avian influenza H5N1 outbreaks in villages in Thailand, January–May 2004 (188 villages of 193 flocks) and July–December 2004 (1,243 villages of 1,492 flocks).

On July 3, 2004, the recurrence of HPAI was confirmed in layer farms in Ayudthaya and Pathumthani Provinces, north of Bangkok. These viruses were characterized as the H5N1 subtype, with genetic sequences similar to the H5N1 isolated in January 2004 (*26*). During P2, HPAI infections were detected in 1,243 villages in 51 provinces (Table 3), which were concentrated in the same 3 regions (Figure 3). From July 3 onward, ≈1–5 cases per day were detected in the first weeks of the epidemic. It reached a peak of 61 cases per day in mid-October 2004 (Figure 2B).

**Figure 4:**
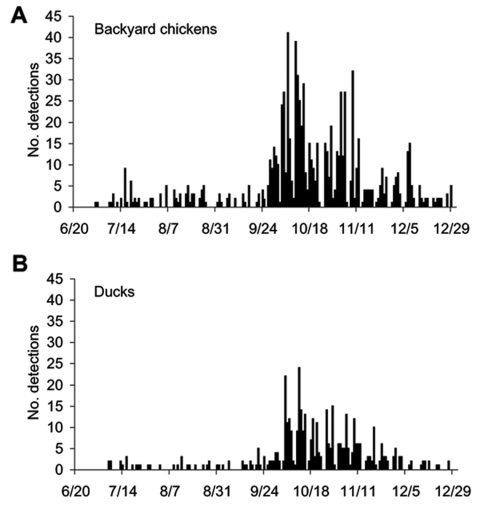
Infected flocks by day of detection and type of poultry, January–May 2004 (panels with "-1" suffix) and July–December 2004 (panels with "-2" suffix). A) Backyard chickens. B) Ducks. C) Broilers. D) Layers.

The geographic distribution of the second wave differs markedly from that of the first wave, and the number of confirmed outbreaks was 8 times higher. Most HPAI outbreaks were found in the Central and Northern Regions where chicken and duck flocks are relatively more abundant. In the Northern Region, 99% of infected flocks were detected in the southern part. Figure 3 shows that HPAI was sporadic in the Southern, the northern part of the Northern, and the Northeastern Regions, which have a lower number and density of poultry populations. Figure 2 shows a dramatic increase in HPAI-positive flocks in January and October 2004, which coincided with the nationwide surveillance programs implemented at that time. Also, the number of infected flocks, particularly of backyard chickens and ducks, increased markedly in these months (Figure 4).

## Type of Poultry Affected

Table 1 shows the various types of poultry in HPAI-positive flocks in 2004. Eighty-three percent of infected flocks were backyard chickens (56%) or ducks (27%); the rest were broilers (6%), layers (5%), quails (2%), and other birds (3%). From field studies in early 2004, ducks were determined to be silent carriers of HPAI virus (*10**,**27*). Accordingly, the proportion of infected ducks diagnosed during P2 markedly increased when compared to the number diagnosed during the early epidemic (P1) because more samples from ducks were submitted to laboratories.

**Figure 5:**
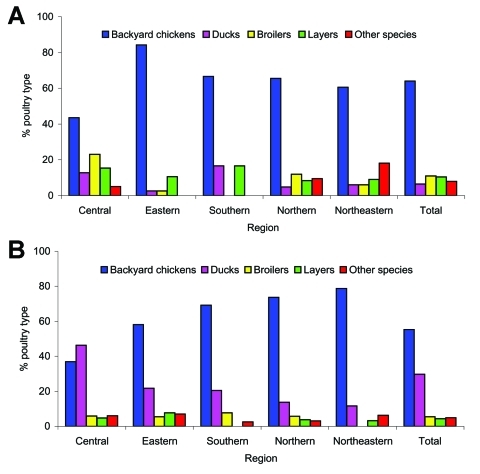
Percentage of main poultry types in infected flocks by region during the 2004 HPAI H5N1 epidemic in Thailand. A) January–May 2004. B) July–December 2004.

Figure 4 shows epidemic curves by species; consistent dissemination of infection was confined to backyard chickens and ducks. Figure 5 illustrates the percentage of HPAI-infected poultry by region. More than 50% of infected flocks were of the backyard type in all regions except the Central Region (≈40% of infected flocks were backyard), which suggests that backyard chickens played a crucial role in the epidemic. However, during P2, 46% of infected flocks in the Central Region were ducks, which shows that they also contributed substantially to the epidemic. Free-grazing ducks are common in the Central Region (Table 1), with its abundance of wetlands and rice paddies. In 102 flocks (6.05%), HPAI was detected in >1 species (mixed farms).

Table 1 also shows cumulative incidence and relative risk (RR) of HPAI outbreaks. RRs of a flock's becoming inflected were 3.7 and 1.3 times higher, respectively, in the Central and Eastern Regions compared to the Northern Region. Moreover, risks for HPAI infection were 5.3, 5.1, 1.5, 32.4, and 2.3 times higher, respectively, in layers, broilers, ducks, quails, and geese compared to backyard chickens.

## Spread to Other Species

In the early epidemic, domestic cats, captive tigers, and leopards also died from H5N1 viruses, which indicates that avian influenza can cross species barriers (*13**,**20**,**25*). In October 2004, the infection of H5N1 viruses was confirmed in captive tigers at Sriracha tiger zoo in Chonburi Province, eastern Thailand (*28*). A total of 147 of 441 tigers kept in the zoo died or were euthanized to prevent possible spread to other zoo animals. Fresh chicken carcasses used to feed the zoo animals, contaminated with HPAI viruses, were considered to be the most plausible source of the infection (*28*).

## Control Measures

### Basic Control Measures

Several measures were taken after the first isolation of HPAI virus in January 2004. Initially, all poultry, their products, feed, bedding, waste, and manure from infected flocks were destroyed immediately by the veterinary authorities. Culling infected birds in each flock was generally completed 1–2 days after the virus was confirmed by virus isolation (confirmatory diagnosis took ≈2–8 days after submission of samples). Meanwhile, a restriction on moving poultry and their products within a 5-km radius around the infected flocks was enforced by DLD inspectors in collaboration with local police, and control checkpoints were temporarily established in these areas. Moreover, infected premises and equipment were cleaned and disinfected.

In July 2004, DLD implemented a series of control measures to enable quick action. Specifically, if the poultry death rate in any facility was >10% within a single day, all birds, their products, and other potentially contaminated materials had to be destroyed without delay. Cloacal swabs of affected flocks were then collected for laboratory confirmation. Subsequently, neighboring flocks were destroyed immediately or quarantined until H5N1 laboratory confirmation. Upon a confirmative laboratory result, quarantined flocks were culled. Furthermore, movement of poultry and their products was restricted within a 1- to 5-km radius around the infected area.

### Preemptive Culling

In January 2004, contiguous flocks were preemptively culled as quickly as possible within a 5-km radius of a confirmed outbreak. After July 2004, preemptive culling was implemented only within a village, within an area of 1 km around an outbreak, or on suspected farms. This new strategy was adopted because the density of poultry flocks decreased after the massive culling during P1. Negative public perception of massive culling was another reason that this strategy was revised.

### Surveillance and Diagnosis

In mid-January 2004, DLD launched a nationwide surveillance program to detect possible HPAI infections in poultry. Cloacal swabs were randomly collected from 4 flocks in each village (5 birds per flock). Swab samples were placed in tubes that contained virus transfer medium; usually 5 swabs were pooled per tube. During P1, >100,000 tubes of swab samples were tested for avian influenza virus. During P2, ≈130,000 tubes of swab samples and 72,000 serum samples were collected for diagnosis.

Swab samples as well as sick or dead bird specimens were submitted to NIAH or regional laboratories. All samples were processed for virus isolation in embryonated chicken eggs (≈1–2 days) (*2*); 2 serial passages in embryonated chicken eggs were performed before a specimen was regarded as negative (≈8 days). In January 2004, the first avian influenza isolate was sent to the University of Hong Kong to identify the virus and serotype hemagglutinin (HA) and neuraminidase (NA) antigens. Thereafter, NIAH itself established the necessary facilities to identify and serotype virus. Furthermore, real-time reverse transcription–polymerase chain reaction analyses for avian influenza were used to detect the virus at all laboratories to reduce the time of diagnosis. Hemagglutination inhibition (HI) test was used to detect antibodies to avian influenza virus in serum samples (*2*).

A nationwide comprehensive surveillance program (known as "x-ray survey") was implemented October 1–31, 2004. The goal of this survey was to detect HPAI infection in any village. In close collaboration among the Ministry of Agriculture and Cooperatives, MOPH, and provincial governors, volunteer public health MOPH workers and DLD livestock workers searched for and reported sick and dead poultry in villages. Through the surveillance program, farmers were also persuaded to report sick or dead poultry in their flocks to authorities. In 2005, x-ray surveys were implemented continuously every 6 months. Moreover, commercial poultry flocks will spend ≈8 days waiting for the results of cloacal swab or blood tests; only if birds are free of the virus will their owners be allowed to move them to slaughterhouses or new areas.

### Other Supportive Measures

A public awareness campaign was started to educate the public on avian influenza and to bolster consumers' confidence that poultry was safe. In addition, the so-called "Big Cleaning Week" was promoted from March 1 to 7, 2004, to encourage relevant parties to be aware of HPAI and to disinfect their facilities, e.g., poultry houses, farm equipment and vehicles, slaughterhouses, and retail markets. Soaps, detergents, alkalis, acids, aldehydes, chlorine, and quaternary ammonium compounds were used as disinfectants. Poultry exhibition and cockfighting were prohibited (since early 2004). A violation of this regulation is subject to fine. Additionally, the practice of allowing ducks to freely graze was discontinued. Because of traditional farming styles, however, these practices are unlikely to change in a short period of time. After an affected flock was culled, a wait of >60 days in broiler farms and >90 days in layer farms and backyard chickens was imposed before a new flock could be established. Farmers must also improve sanitary measures in their farms to meet DLD's requirements.

## Consequences of Epidemic

In early 2004, lack of information and communication with regard to HPAI caused the public to lose confidence in poultry products. The decrease in domestic consumption and bans on Thai poultry products by importing countries damaged the poultry industry. In addition, H5N1 virus from poultry caused 17 human cases with 12 deaths in 12 provinces (Figures 2 and 3) (*15**,**22*).

The Thai government used a stamping-out policy to control HPAI outbreaks and compensated farmers for their losses. According to the Animal Epidemic Act, farmers are entitled to compensation of 75% of the value of animals that are destroyed. However, 100% compensation was provided during P1 because the epidemic was widespread and devastating to Thai farmers; compensation was reduced to 75% during P2. Compensation per bird was (in US dollars) $0.38–$65, depending on the type of poultry ($0.38 for quail; $1.13 for broiler; $2 for meat duck; $2.25 for backyard chicken; $3.5 for layer chicken, layer duck, or goose; $7.25 for turkey; and $65 for ostrich).

Approximately 62 million birds were either killed by H5N1 viruses or culled for disease control and animal welfare reasons. The government allocated a budget of ≈5.3 billion Thai baht (US $132.5 million) for direct compensation to affected farmers (*29*). All costs of implemented basic measures were covered by the government. As of March 2004, the HPAI epidemic had an estimated effect on the national gross domestic product of 0.39%. These losses amount to 25.24 billion Thai baht (US $631 million) (*30*).

## Conclusions

### Detection and Early Distribution of HPAI Virus

Epidemiologic data from the early epidemic indicate that the period between the introduction of the virus into Thailand and its conclusive identification was too long. The route of virus introduction could not be traced. Also the delay between primary infection, first diagnosis, and finding the initial case allowed widespread dissemination of the virus and contributed to the large scale of the epidemic (*31**,**32*). Early warning, early detection, and early response are essential to prevent and control HPAI. In view of potential public health implications of HPAI, notifying and collaborating with public health authorities is equally important.

### Geographic Regions, Affected Species, and Incidence

The epidemic differed by region. The Central and Northern Regions contained 82% of the total outbreaks (Table 1). Infections were prominent in backyard chicken flocks in the southern part of the Northern Region and in free-grazing ducks in the area adjacent to the Central Region. Backyard chickens and free-grazing ducks played essential roles as H5N1 hosts (Figure 4); 83% of confirmed flocks were backyard chickens or ducks. Because of improved surveillance during P2, disease detections increased markedly (Figure 2). The difficulty of clinically detecting HPAI in ducks (*27**,**33*) and free-ranging backyard chickens and ducks also made controlling the disease difficult.

The 2004 cumulative incidence and RR also show a higher number of detections in the Central, Northern, and Eastern Regions relative to other parts of Thailand (Table 1). This finding reflects that the high density of poultry, the local geography (e.g., wetlands, water reservoirs, and rice paddies), and farming practice in these regions might be risk factors for outbreaks. Other studies showed a strong association between free-grazing duck populations and the practice of free-grazing farming with spread of the virus in the Central Region (M. Gilbert et al., pers. comm.). In our exploratory analysis, the RR for HPAI infection in Thai poultry production could not be clearly elucidated. The RR for HPAI infection was high in broilers, layers, quails, geese, and ducks compared to backyard chickens (Table 1). The number of detections in these types of poultry increased substantially in backyard chickens and ducks when national surveillance was implemented in January and October 2004. This observation suggests that when larger-scale farmers observed suspected cases in layer and broiler farms, they immediately reported them to local authorities, encouraged by the compensation that they received. In contrast, small farmers most likely did not report their few dead poultry. Consequently, the number of outbreaks in small farmers may have been underestimated. Additionally, size of flock may be a confounding factor in the higher risk for infection in broiler, layer, and quail flocks (*34**,**35*).

### Course of the Epidemic

The epidemic curve during P1 shows a steep rise in the first week; detections decreased sharply after control measures were taken (Figure 2A). In the early epidemic, samples of culled flocks were not tested during massive culling. Undoubtedly, the quantity of infected flocks was underestimated, thus obscuring the effectiveness of control measures to stem the outbreaks. High numbers of HPAI detections coincided with low temperatures in Thailand from October to February, when wild birds from central and northern Asia migrate into Thailand (*7*). Therefore, seasonal conditions and bird migration might have contributed to the introduction of HPAI virus. Furthermore, the lower temperature supports survival of the virus in the environment and facilitates transmission (33). In addition, several festivals, which are associated with raising, selling, and transporting poultry, occurred around the end of the year. Illegal transportation and cockfighting may have worsened the HPAI situation.

### Effectiveness of Control Measures

Because of differences and changes to control measures and surveillance programs during P1 and P2, HPAI outbreak data are difficult to compare. The start of the outbreak was an emergency period, during which epidemiologic data could not be effectively or completely collected. However, our results indicate that although several measures were implemented in 2004, the epidemic could not be controlled. HPAI outbreaks can be controlled rapidly with highly restrictive measures by totally depopulating all poultry in the entire areas in some countries (*6**,**32**,**36*). However, given that HPAI was widespread in all parts of Thailand, total depopulation was not a practical option. But a combination of depopulation with improved early detection and response practiced during P2, combined with the culling rigor practiced during P1, may be a realistic option.

The Thai epidemic shows that the virus continues to circulate in the country. The immediate challenge is, therefore, to control avian influenza in free-ranging animals in rural areas, particularly in backyard chickens and free-grazing ducks. However, control of outbreaks in these types of poultry is difficult because of traditional farming practices. Control could be achieved by improving biosecurity of poultry farms and changing farming practices (*6**,**36**,**37*). Meanwhile, educating farmers and staff on early detection and the basic concepts of biosecurity may be the most critical way to eliminate avian influenza virus (*32*).

Since January 2004, a stamping-out policy has been used to control avian influenza outbreaks in Thailand; vaccination has been not allowed. According to the Office International des Épizooties Terrestrial Code 2005, 2 broad vaccination strategies exist, inactivated whole avian influenza viruses and hemagglutinin expression–based vaccines. Thus, vaccination may be worthwhile to consider as an additional control measure (*36*). Vaccination significantly reduces excretion of viruses (*38**,**39*), which may reduce viral load in the environment and decrease the risk for human exposure. However, HPAI infection could become endemic if vaccination is not managed appropriately (*40*).

Early detection of all cases was essential to rapidly implement control measures. Meanwhile, comprehensive veterinary surveillance and long-term control measures are required (*11*). The success of HPAI elimination, therefore, depends on a collaboration of all stakeholders, including farmers, industries, veterinarians, public health authorities, academic institutions, media, and the government (*36**,**37*).
